# Exploration of the Association Between Anterior Disc Displacement Without Reduction and Electromyographic Activity of Masseter Muscle Based on Needle Electromyography

**DOI:** 10.3390/jcm15135148

**Published:** 2026-07-02

**Authors:** Chu-Han Zhang, Jia-Ying He, Min-Han Li, Bian Wang, Shan Jin, Miao Chen, Chi Yang, Zhi-Gui Ma

**Affiliations:** 1 Department of Oral Surgery, Shanghai Ninth People’s Hospital, Shanghai Jiao Tong University School of Medicine, College of Stomatology, Shanghai Jiao Tong University National Center for Stomatology, National Clinical Research Center for Oral Diseases, Shanghai Key Laboratory of Stomatology, Shanghai Research Institute of Stomatology, Shanghai 200011, China; zhangchuhan0715@163.com (C.-H.Z.); jiay_he@163.com (J.-Y.H.); liminhanliminhan@163.com (M.-H.L.); jins2721@sh9hospital.org.cn (S.J.); zerochen0828@163.com (M.C.); 2 Department of Assisted Reproduction, Shanghai Ninth People’s Hospital, Shanghai Jiao Tong University School of Medicine, Shanghai; College of Stomatology, Shanghai Jiao Tong University, National Center for Stomatology, Shanghai; National Clinical Research Center for Oral Diseases, Shanghai Key Laboratory of Stomatology, Shanghai 200011, China; 15216708276@126.com; 3 Department of Oral Surgery, Shanghai Ninth People’s Hospital, Shanghai Jiao Tong University School of Medicine, College of Stomatology, Shanghai Jiao Tong University, National Center for Stomatology, National Clinical Research Center for Oral Diseases; Shanghai Key Laboratory of Stomatology, Shanghai Research Institute of Stomatology, Research Unit of Oral and Maxillofacial Regenerative Medicine, Chinese Academy of Medical Sciences, Shanghai 200011, China

**Keywords:** anterior disc displacement without reduction, temporomandibular internal derangement, masseter muscle, needle electromyography

## Abstract

**Background**: Patients with temporomandibular internal derangement, especially anterior disc displacement without reduction (ADDwoR), often present with limitations in masticatory function. The aim of this study was to explore the association between ADDwoR and electromyographic activity of the masseter muscle using needle electromyography. **Methods**: A total of 137 participants, including 64 controls with normal TMJs and 73 with bilateral ADDwoR, underwent needle electromyography of the masseter muscle. Abnormal electromyographic activity was compared between groups, and logistic regression analyses were performed to examine the associations between TMJ characteristics and electrophysiological abnormalities. **Results**: Patients with ADDwoR showed a significantly higher prevalence of abnormal masseter electromyographic activity than controls. Compared with the control group, MUPs in the ADDwoR group had longer duration, higher amplitude, and larger area. Multivariable logistic regression showed that ADDwoR was associated with the presence of these abnormalities. Exploratory subgroup analyses suggested possible associations with condylar resorption and disc morphology, though these findings should be interpreted with caution. **Conclusions**: Bilateral ADDwoR is associated with altered masseter muscle MUPs and a higher prevalence of abnormal needle EMG findings. Exploratory analyses suggest possible associations with condylar resorption and disc morphology, but these results require confirmation in larger studies. These findings suggest that both TMJ structural assessment and masticatory muscle functional evaluation may be useful in the clinical assessment of patients with ADDwoR.

## 1. Introduction

Temporomandibular disorders (TMDs) represent a group of musculoskeletal conditions affecting the temporomandibular joint (TMJ), masticatory muscles, and related structures [1]. Their global prevalence is approximately 34% in the general population, with some estimates rising to 44% [2,3]. Anterior disc displacement (ADD) is one of the most common forms of internal derangement and is defined by anterior displacement of the articular disc relative to the condyle [4,5]. Based on whether the disc returns to its normal disc-condyle relationship during mouth opening, ADD is classified as anterior disc displacement with reduction (ADDwR) or without reduction (ADDwoR) [6]. The diagnosis of ADD subtypes follows the Diagnostic Criteria for Temporomandibular Disorders (DC/TMD) [7]. Magnetic resonance imaging (MRI) is the gold standard for assessing disc position and morphology, and cone-beam computed tomography (CBCT) is also valuable for evaluating condylar bone changes. Advances in digital technology, including jaw tracking devices such as Modjaw, have further enabled the real-time analysis of mandibular kinematics and functional occlusion. ADDwoR may cause limited mouth opening, chronic joint pain, condylar resorption, and craniofacial deformity [8]. Moreover, some patients also report masticatory muscle pain, reduced chewing efficiency, and difficulty with mastication, which may be accompanied by mandibular movement dysfunction [9,10]. These findings suggest that TMDs may be related to changes in masticatory muscle function, but the relationship remains poorly understood.

In recent years, more attention has been given to the association between the masticatory muscles and TMJ. Morphological studies have shown that masseter muscle thickness and cross-sectional area were correlated with TMJ structure in patients with skeletal Class II malocclusion [11]. In addition, patients with bilateral idiopathic condylar resorption (ICR) exhibit smaller masseter and temporalis muscle volumes compared with normal individuals, indicating masticatory muscle atrophy in ICR patients [12]. These findings indicate that masticatory muscle morphology is associated with TMJ conditions. However, TMD is a multifactorial disorder, and its symptoms are not determined by structural changes alone. Central pain modulation, psychosocial factors, and parafunctional habits may also contribute to pain expression and muscle activity [13,14]. Therefore, the relationship between TMJ pathology and masticatory muscle function should be interpreted within a broader functional framework.

Electromyography (EMG) is commonly used to detect abnormal muscle activity, analyze electrical signals, and assess muscle function, and it plays a crucial role in the diagnosis and management of oral myofunctional disorders [15]. Surface electromyography (sEMG) has been widely used to evaluate muscle activity because of its noninvasive and convenient features, and meta-analyses have reported that patients with TMD show higher sEMG activity at rest but lower activity during maximal contraction [16,17]. However, sEMG also has limitations. Its sensitivity may be affected by hair in the temporal region, and it is less suitable for assessing deep muscle activity, which reduces its value for evaluating motor unit potentials and neuromuscular disorders [18,19]. In addition, previous studies have suggested that disturbances in stomatognathic muscle activity were multidimensional and may also be associated with neurogenic mechanisms and broader functional disorders, highlighting the need for neurophysiological assessment of masticatory muscles in TMD [20]. However, whether ADDwoR is associated with abnormal motor unit level activity in the masticatory muscles remains largely unexplored.

Needle electromyography (nEMG) is a technique that uses a fine electrode inserted directly into the muscle, allowing recording of electrical signals from individual motor units and evaluation of muscle function [21]. Studies have demonstrated that peripheral orofacial alterations, such as orthodontic tooth movement and occlusal changes, can induce neuroplastic changes in the sensorimotor cortex that controls the masticatory muscles [22]. This suggests that chronic disc displacement, as a persistent source of altered proprioceptive input from temporomandibular joint mechanoreceptors, may similarly drive neuroplastic mechanisms, ultimately affecting motor unit behavior. These changes can be specifically detected by nEMG as alterations in motor unit potential (MUP) duration, amplitude, area, and recruitment patterns. Thus, nEMG is well-suited to investigate masticatory muscle neurophysiology in ADDwoR. The masseter muscle was selected as the target muscle in the present study because it plays a major role in mandibular elevation and bite force generation, and it is also readily accessible for access with a needle electrode. In addition, our previous research and some other studies have suggested that the masseter was more consistently affected in TMD patients [23].

The study aimed to compare nEMG parameters and the prevalence of EMG abnormalities between patients with bilateral ADDwoR and controls, to evaluate the association between the severity of condylar resorption and EMG abnormalities, and to explore whether different disc morphology types are related to EMG abnormalities. The findings are intended to contribute to a more comprehensive understanding of the relationship between ADDwoR and masticatory neuromuscular function, with implications for the integrated clinical management of patients. The null hypothesis was that there would be no significant differences in abnormal MUP parameters or the prevalence of EMG abnormalities between patients with and without ADDwoR, and that condylar resorption and disc deformation would show no association with EMG alterations.

## 2. Materials and Methods

### 2.1. Participants

The study was approved by the Human Research Ethics Committee of Shanghai Ninth People’s Hospital, Shanghai Jiao Tong University School of Medicine (SH9H-2024-T278-1), and was conducted in accordance with the Declaration of Helsinki and STROBE guidelines. The study included two groups of patients with ADDwoR and control participants without ADDwoR. All participants and their parents provided informed consent and agreed to participate in the study.

In total, 173 patients visited the TMJ-Orthodontic Clinic of the Department of Oral Surgery at Shanghai Ninth People’s Hospital and received nEMG examinations over 2 years. Of the 173 participants, 36 were excluded, including 12 with unilateral ADDwoR, 10 with ADDwR, 12 with TMJ surgery history and 2 with a history of anxiety and were receiving medication. The final study sample consisted of 137 participants. The ADDwoR group comprised 73 patients who met the following inclusion criteria: (1) patients diagnosed with bilateral ADDwoR according to Diagnostic Criteria for Temporomandibular Disorders (DC/TMD) and confirmed by MRI; (2) TMJ clinical symptoms, including noises, joint pain or limited mouth opening. The control group consisted of a clinic-based sample of 64 participants who visited the same clinic primarily for orthodontic consultation, had a normal disc-condyle relationship confirmed by MRI, had smooth and intact condylar morphology, and had no current or previous symptoms of TMD. Exclusion criteria were as follows: (1) a history of craniomaxillofacial trauma, jaw fracture, orthognathic surgery, infection, or other clinically significant pathologies affecting the growth of the craniofacial skeleton; (2) prior TMJ treatment, including splint therapy, arthrocentesis, or TMJ surgery; (3) diagnosed neurological or neuromuscular disease; (4) systemic disease such as arthritis and immune disorders; and (5) psychiatric or psychological disorders or long-term use of psychotropic medication.

### 2.2. MRI Examination of Condyle and Disc Morphology

All participants underwent MRI examination using a 1.5-Tesla imager (Signa; General Electric, Milwaukee, WI, USA). Proton density-weighted and T2-weighted images were obtained in both closed-mouth and open-mouth positions. In the closed-mouth position, anterior disc displacement was defined as a disc position exceeding +15° from the 12 o’clock position of the condyle. If the disc did not return to a normal position during mouth opening, it was classified as ADDwoR [24].

**Figure 1:**
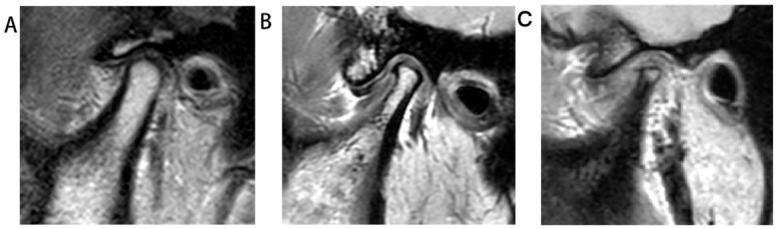
Examples of subgrouping patients using magnetic resonance imaging. (**A**) Normal condyle. (**B**) ADDwoR with mild condylar resorption. (**C**) ADDwoR with severe condylar resorption.

According to Hu et al. [25], disc morphology in ADDwoR was classified into three types based on disc shape and length: type III: moderate folded, U-shaped or V-shaped disc with sufficient length to cover the condylar head; type IV: folded and shortened disc with inadequate length to cover the condylar head; type V: severely folded, biconvex or rounded configuration. Condylar resorption (CR) was assessed according to the condyle condition, and the ADDwoR group was further divided into mild and severe resorption subgroups following Yang’s classification (Figure 1). Mild CR was defined as localized resorption or CR with mildly reduced height, whereas severe CR was defined as a small condyle, severe height reduction, loss of integrity of cortical bone, or complete resorption [26]. Accordingly, disc morphology and condylar resorption (CR) were converted into ordinal scores. Type III, type IV, and type V were assigned scores of 1, 2, and 3, respectively, and mild and severe CR were assigned scores of 1 and 2. To account for bilateral TMJ cases, the scores from the left and right sides were averaged to generate the composite score.

Two TMJ specialists who were blinded to the EMG results independently analyzed all MRI images (Z.M. and C.Y.). Prior to the evaluation, they independently assessed a separate set of 20 pilot scans to calibrate their understanding of the classification criteria and to discuss any ambiguous cases. Inter-rater reliability for the MRI diagnosis of ADDwoR, disc morphology and condylar resorption grading was assessed using Cohen’s kappa coefficient. When disagreements occurred, the final diagnosis was reached by consensus through discussion.

### 2.3. Electromyography (EMG) Procedure and Diagnosis

nEMG recordings were performed at room temperature using the Neuropack X1 system (Nihon Kohden Corporation, Tokyo, Japan), with a band-pass filter set at 20 Hz–10 kHz and a sweep speed of 10 ms/division. Participants were placed in the supine position, and their skin over the insertion site was cleansed with 75% alcohol before electrode insertion. A concentric needle electrode was inserted into the most prominent area of the masseter to a depth of approximately 10–15 mm, and the reference electrode was placed on the tendon. A short stabilization period was allowed before any recording began. After evaluation of spontaneous activity, motor unit potentials (MUPs) were recorded during low-level contraction of the masseter muscle (approximately 10% of maximal voluntary contraction), followed by needle movement through the muscle to sample additional MUPs. For each MUP, rise time, slope, amplitude (μV), duration (ms), and area were recorded using on-screen cursors. At least 20 MUPs were collected from each participant according to the standard protocol, and the mean value was used for analysis.

**Figure 2:**
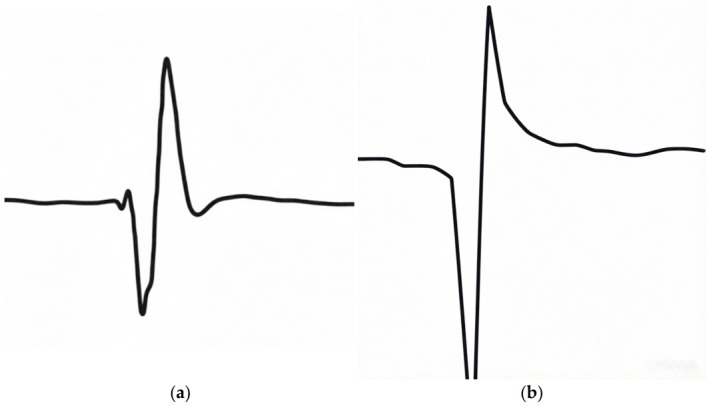
Representative examples of motor unit potentials (MUPs) recorded from the masseter muscle. (**a**) Normal MUP with normal duration and amplitude; (**b**) Abnormal MUP showing prolonged duration and increased amplitude.

Participants were then instructed to perform maximal voluntary contraction (MVC) for 20 s to record motor unit recruitment and analyze the interference pattern using the automatic analysis program. Electrophysiological abnormalities were defined as increased MUP duration (>10 ms) and high amplitude (>2 mV) based on the mean values of bilateral TMJs, or reduced motor unit recruitment, according to standard clinical criteria [27,28]. Representative examples of normal and abnormal MUP waveforms are shown in Figure 2.

All diagnoses were made by an experienced neurophysiologist using established methods [29]. MUP feature values were collected and evaluated by the same specialist, who was blinded to the TMJ diagnoses and MRI findings. Each measurement was repeated three times, and the average value was used as the final result. To assess intra-observer reliability, the same neurophysiologist re-evaluated a subset of 20 randomly selected nEMG recordings after an interval of at least four weeks.

### 2.4. Statistical Analysis

Complete-case analysis was performed for the statistical analyses using IBM SPSS Statistics software version 27.0 (Chicago, IL, USA). Disordered electromyographic activity in the masseter muscle was defined by the presence of electrophysiological abnormalities. Categorical variables were presented as frequencies and percentages and were analyzed using the chi-square test. The Mann–Whitney U test and the independent *t*-test were used for comparisons of continuous variables between the two groups. Three separate multivariable logistic regression analyses were conducted to examine the associations between TMJ structural characteristics and electrophysiological abnormalities. All models were adjusted for age and sex. Model 1 incorporated disc position (control vs. ADDwoR); Model 2 included the composite score for condylar resorption; and Model 3 included the disc morphology score. For bilateral TMJ cases, the scores from the left and right sides were averaged to obtain a single participant-level score for the primary analyses. In addition, supplementary analyses were performed with the original subtype classifications of condylar resorption and disc morphology. When the two sides differed, the more severe type or grade was selected. Sensitivity analyses were also performed among female participants for the three logistic regression models due to the distribution of sex. Results were reported as odds ratios (ORs) with 95% confidence intervals (CIs) and corresponding *p* values. Effect sizes were reported as Cohen’s d for *t*-tests, Cramér’s V for the χ^2^ test, and odds ratios for logistic regression [30]. A *p* value of <0.05 was considered statistically significant.

Based on a prior pilot observation indicating an abnormal EMG prevalence of approximately 10% in healthy controls and 35% in ADDwoR patients, a medium effect size was assumed for sample size calculation according to recent guidelines specific to dentistry [30]. Using G*Power 3.1 for a chi-square test of two independent proportions, with a two-sided significance level of 0.05, a statistical power of 80%, and equal group sizes, the required sample size was calculated to be at least 55 participants per group. The final enrolled sample of 64 controls and 73 ADDwoR patients exceeded this requirement. The events per variable (EPV) ratio was calculated to assess the adequacy of the logistic regression models. EPV was defined as the number of events divided by the number of candidate predictor variables included in the logistic regression model, with an EPV ≥ 10 generally considered acceptable [31].

## 3. Results

73 patients diagnosed with bilateral ADDwoR were included in the study to investigate the association between TMD and abnormal electrical activities of the masticatory muscle, with an average age of 23.96 years old (SD = 5.68). A total of 64 patients with normal TMJs were included as the control group, with a mean age of 23.31 years (SD = 5.83). No significant difference in age was observed between the two groups, whereas the ADDwoR group had a significantly higher proportion of females (93.2%; Table 1). Patients in the ADDwoR group were further classified into subgroups according to disc and condylar characteristics. As no significant differences were found between the left and right TMJs for continuous variables in bilateral cases based on paired *t*-tests, data from both sides were combined for analysis. Inter-rater agreement was excellent for ADDwoR diagnosis (Cohen’s κ = 0.82, 95% CI: 0.723–0.911), condylar resorption grading (κ = 0.79, 95% CI: 0.684–0.883), and disc morphology classification (weighted κ = 0.88, 95% CI: 0.805–0.948), all with *p* < 0.001, and initial disagreement rates of 8.76%, 10.21%, and 9.49%, respectively. In addition, a reliability analysis on 20 randomly selected subjects showed good agreement (Cohen’s κ = 0.85).

A participant flow diagram is shown in Supplementary Figure S1.

### 3.1. The Electrical Activity of the Masseter Muscle

**Figure 3:**
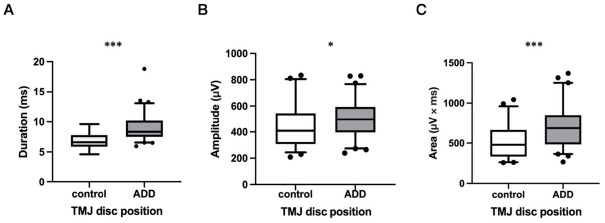
Box plots of motor unit potential parameters in the masseter muscle. (**A**) MUP duration (ms), (**B**) MUP amplitude (μV), (**C**) MUP area (μV × ms). Box, IQR, 25–75%; line, median; whisker interval, 5–95%; * *p* < 0.05; *** *p* < 0.001. Individual data points beyond the whiskers are plotted as dots.

The χ^2^ test analysis showed that abnormal electromyographic activity of the masseter muscle was associated with TMJ ADDwoR (Table 2). The frequency of abnormal MUP findings was significantly higher in the ADDwoR group than in the control group (χ^2^ = 14.218, *p* < 0.001, Cramer’s V = 0.37). A summary of the results obtained using *t* tests for MUP amplitude, area, and duration is shown in Figure 3. Compared with controls, MUPs in ADDwoR patients demonstrated longer duration, higher amplitude, and larger area (Duration: *p* < 0.001, Cohen’s d = 1.82; Amplitude: *p* = 0.011, Cohen’s d = 0.44; Area: *p* < 0.001, Cohen’s d = 0.84). The effect sizes were large for duration and area, and moderate for amplitude. During maximal voluntary contraction, a simplified recruitment pattern was more frequently observed in the ADDwoR group.

### 3.2. Association Between Electromyographic Activity and TMJ Characteristics

The results of the multivariable logistic regression analysis are presented in Table 3. The EPV values were 11 for the three models.

After adjustment for sex and age, ADDwoR was associated with higher odds of masseter muscle EMG abnormalities compared with normal TMJs (OR = 9.52, indicating 9.52-fold higher odds). In exploratory analyses with composite scores, the score for condylar resorption was associated with increased odds of EMG abnormalities (aOR = 2.112), suggesting that greater overall resorption severity may be related to a higher risk (Table 4). However, the composite score for disc morphology did not reach statistical significance (Table 5). The results based on the original subtype classifications were presented in Supplementary Tables S1 and S2. In those analyses, condylar resorption (mild: OR = 9.482; severe: OR = 9.571) and disc morphology (type III: OR = 10.815; type IV: OR = 9.971; type V: OR = 7.080) showed a positive association with EMG abnormalities compared with normal TMJs, but the confidence intervals for the disc morphology subtypes were wide and overlapping, which may be attributable to the limited sample size in each subgroup, especially for type V. Accordingly, a linear trend across morphological subtypes could not be clearly established. The true relationship remains uncertain, and studies with larger cohorts are warranted for further investigation.

Female-only sensitivity analyses yielded results consistent with the primary analyses and are presented in Supplementary Tables S3–S5. Results after multiple-comparison correction are provided in the Supplementary Tables S6 and S7 and were consistent with the main findings.

## 4. Discussion

The study found patients with ADDwoR exhibited significantly abnormal electromyographic findings in the masseter muscle, including prolonged MUP duration, increased amplitude, and larger area, compared with the control group. These findings indicate that ADDwoR is associated with altered electrophysiological activity of the masticatory muscles. Further analyses indicated that condylar resorption and disc deformation were also related to the MUP alterations after adjustment for age and sex. The main results supported the associations between electrophysiological changes in the masseter muscle and ADDwoR, suggesting that both TMJ condition and muscle function should be considered in clinical evaluation.

Patients with ADD, particularly ADDwoR, commonly complain of limitations in masticatory function [32] and present for consultation due to spontaneous pain, tenderness, or movement-related pain in the TMJ region and surrounding muscles, mandibular movement dysfunction, and clicking sounds. These clinical manifestations suggest that TMJ ADD is not merely a structural abnormality but may also impair the functional behavior of the masticatory system. Because the TMJ and masticatory muscles function as an integrated system, ADDwoR-related changes in joint structure, position, loading, and mandibular movement may affect masseter muscle activation and motor unit recruitment. Previous studies have primarily employed sEMG to assess the masticatory muscles in TMD patients. Although the diagnostic and prognostic value of electromyography remains controversial, these studies generally indicate that TMD is associated with abnormal electrical activity in the masticatory muscles [33,34]. However, sEMG reflects global muscle activity and does not allow evaluation of individual motor unit potentials or detection of neurogenic remodeling. For this reason, we employed nEMG in the present study. In contrast to sEMG, nEMG records the electrical activity of individual motor units via a needle electrode inserted into the muscle, thereby providing a more detailed assessment of muscle and motor unit function [16].

In this study, patients with ADDwoR exhibited significantly prolonged MUP duration, increased amplitude, and area compared with comparators. In routine electromyographic interpretation, such MUP changes are often considered compatible with chronic neurogenic remodeling. Multivariable logistic regression analysis also showed that ADDwoR was associated with electrophysiological abnormalities in the masseter muscle. Although our findings do not establish the presence of a primary neuropathic lesion, they suggest that ADDwoR may be accompanied by alterations in motor unit behaviors in the masticatory muscles. The mechanisms linking ADDwoR to these MUP changes are likely multifactorial and may involve both biomechanical and neurophysiological pathways [29,35]. One possible explanation is that chronic disc displacement and condyle position may alter proprioceptive inputs from TMJ and periodontal receptors. In addition, ADDwoR may affect occlusion relationships and thereby contribute to biomechanical changes [36,37]. Malocclusion, such as unilateral posterior crossbite or deep bite, has also been implicated as a possible risk factor for disc displacement [38], which further supports the multifactorial nature of TMD and suggests that occlusal assessment may be relevant in ADDwoR patients. These changes may in turn influence motor control within the trigeminal motor system and may be associated with compensatory alterations in motor unit recruitment. In this context, the prolonged and enlarged MUPs observed in our study may be consistent with a secondary neurophysiological adaptation. Similar explanations have been proposed in temporomandibular joint disorders, and Isberg et al. reported that disc displacement can directly trigger masticatory muscle electromyographic activity via an arthrokinetic reflex [39]. However, the electrical activity of the masseter muscle is a complex compound process, and the mechanisms underlying the association between ADDwoR and the observed MUP changes remain unclear.

At the same time, other factors may also contribute to the observed findings, and the EMG abnormalities may reflect functional adaptation. Patients with ADDwoR frequently experience pain, masticatory dysfunction, and altered occlusal loading, which may give rise to a different set of muscle adaptations. These adaptive changes, probably related to prolonged overuse, such as bruxism or clenching, pain-related protective muscle inhibition, and load avoidance, may ultimately manifest as characteristic changes in MUP features. These factors may act independently or in combination with joint-related proprioceptive dysfunction, and because we did not systematically adjust for these potential confounders, we cannot rule out the possibility that the observed association may be influenced by residual confounding. Therefore, while the present results support a possible association between ADDwoR and chronic neurogenic-like changes in the masseter muscle, the underlying mechanism remains speculative, and future studies incorporating nerve conduction studies, quantitative sensory testing, and prospective control of confounders are needed to clarify the pathophysiology.

Our subgroup analyses provide additional, though exploratory, support for this interpretation. Condylar resorption was associated with higher odds of EMG abnormalities, suggesting that structural degeneration of the condyle may be linked to altered masseter muscle function. Disc morphology was also associated with abnormal EMG findings, with the ORs varying across subtypes. However, the confidence intervals were wide, particularly in the smaller groups, and the composite score analysis did not reach statistical significance. Therefore, a dose–response relationship still cannot be inferred from the observed numerical trend. Collectively, these findings suggest that ADDwoR and accompanied structural changes, including condylar resorption and disc deformation, were associated with masticatory dysregulation, and that the presence of these morphological abnormalities may warrant attention to masticatory muscle function, but the lack of a clear dose–response gradient and the wide confidence intervals indicate that further studies with larger samples are needed to confirm the nature of these associations.

The present findings suggested that ADDwoR was associated with electrophysiological abnormalities in the masseter muscle. This supports the integrated view of joint and masticatory muscles in TMD patients. From a clinical perspective, when evaluating ADDwoR patients, especially those with condylar resorption or advanced disc deformation, clinicians should be aware of the potential masticatory muscle electrophysiological abnormalities, even in the absence of pronounced muscle pain, which contributes to a more thorough analysis of the disease status. nEMG may provide supplementary information at the motor unit level and may help clinicians better characterize the functional status of the masticatory system in ADDwoR. An assessment of muscle function together with objective internal derangements of the TMJ may facilitate more comprehensive management of ADDwoR patients. However, because this was a retrospective observational study and the present data do not allow conclusions regarding causality, further prospective cohort studies with larger and more balanced samples are needed to clarify the temporal relationship between ADDwoR and masseter muscle electrophysiological changes. In addition, longitudinal studies are needed to determine whether treatment of ADDwoR, such as disc repositioning surgery or occlusal splint therapy, can normalize masseter muscle EMG patterns over time.

However, several limitations of the study should be acknowledged. As a retrospective cross-sectional study, the definitive causal relationship between the two factors cannot be established. In addition, the sample size is relatively modest, especially for subgroup analyses of disc morphology, which reduces precision and may lead to unstable estimates, and the results still need to be interpreted with caution. Potential selection bias should also be considered, as the control group was recruited from orthodontic consultation patients in a single-center and may differ from the general population, which may limit the generalizability of the findings to broader populations. The markedly female-skewed ADDwoR group also limits extrapolation to male patients. In addition, several potential confounders, including bruxism, pain intensity, symptom duration, occlusal status, or psychological factors such as stress and central sensitization, were not systematically assessed in the study. These variables might independently affect masticatory muscle activity and partly influence the observed associations. Additionally, we considered only qualitative EMG findings and quantitative MUP values, excluding other electrophysiological indicators such as blink reflex and nerve conduction velocity, as well as additional clinical indicators of ADDwoR like pain and occlusal position. The diagnostic criteria for neurogenic abnormality relied on general clinical standards, but masseter-specific normative values remain limited, and needle insertion itself may have induced minor local muscle responses. Though the thresholds are widely used in clinical practice to identify chronic neurogenic changes, future studies with masseter-specific reference values may help refine the diagnostic accuracy. We also acknowledge that the classification of condylar resorption was qualitative, which may have introduced some subjective bias despite standardized criteria. Future studies should use validated morphometric indices to assess condylar resorption more accurately. Finally, the composite scores were used to integrate bilateral TMJ information. Although this provides a summary of bilateral involvement, it may not fully reflect differences across severity levels. The categorical analyses are shown in the Supplementary Material as sensitivity analyses. Future studies with larger samples and more detailed analytical strategies would be valuable to better address these methodological issues and to confirm the robustness of our findings.

In summary, ADDwoR was associated with abnormal electromyographic activity in the masseter muscle. While no single mechanism can explain all observed MUP alterations, the evidence supports the clinical importance of evaluating both TMJ structure and masticatory muscle function in patients with ADDwoR.

## 5. Conclusions

Overall, this study suggests that bilateral ADDwoR is associated with changes in masseter muscle MUPs, including prolonged duration, increased amplitude, and increased area. These electrophysiological findings may reflect chronic neurogenic-like changes in the masticatory muscles. In addition, condylar resorption and disc morphological deformation were associated with these MUP abnormalities. These findings indicate that structural TMJ assessment and functional evaluation of the masticatory muscles should be considered together in the clinical evaluation of patients with ADDwoR. However, the underlying mechanisms remain uncertain, and future prospective studies with larger samples and more comprehensive control of confounding factors are needed to clarify these associations.

## Figures and Tables

**Table 1:** Participant characteristics (n = 137).

Variables	ADDwoR (73)	Control (64)	*p* Value
Sex, female (%)	69 (93.2%)	47 (73.4%)	<0.001 ***
Age (y)	23.96 ± 5.68	23.31 ± 5.83	0.513
EMG abnormality, n (%)	27 (37.1%)	6 (9.38%)	<0.001 ***
Duration (ms)	8.92 ± 2.13	6.51 ± 1.39	<0.001 ***
Amplitude (μV)	498.53 ± 136.63	434.41 ± 154.40	0.011 *
Area (μV × ms)	685.40 ± 244.27	495.28 ± 206.38	<0.001 ***
Condylar resorption (n = condyles)			
Mild	88	—	
Severe	58	—	
Condylar resorption score	1.38 ± 0.47		
Disc morphology (n = condyles)			
III	68	—	
IV	55	—	
V	23	—	
Disc morphology score	1.71 ± 0.74		

* *p* < 0.05; *** *p* < 0.001. ADDwoR, anterior disc displacement without reduction; EMG, electromyogram. *p* values were from independent *t*-test or χ^2^ test. Benjamini–Hochberg false discovery rate (FDR) correction was applied for multiple comparisons and the adjusted significance level was set at q < 0.05. All comparisons except age remained significant after correction (Supplementary Table S7).

**Table 2:** Correlation of abnormal electromyographic activity with TMJ ADD.

	Electromyography Condition			
Group	Normal	Abnormal	χ^2^	*p* Value
Control (64)	58	6	14.218	<0.001
ADDwoR (73)	46	27		
Total (137)	104	33		

ADDwoR, anterior disc displacement without reduction.

**Table 3:** Correlation of disc position with electrophysiological abnormalities.

	Electromyography Condition			
Characteristic	Crude OR (95%CI)	*p* Value	Adjusted OR (95%CI)	*p* Value
Age (y)	1.027 (0.958–1.102)	0.450	1.033 (0.958–1.115)	0.397
Sex				
Male	1.00 (reference)	0.358	1.00 (reference)	0.028 *
Female	1.597 (0.589–4.333)		4.345 (1.171–16.116)	
Disc position				
Control	1.00 (reference)	<0.001 ***	1.00 (reference)	<0.001 ***
ADDwoR	5.674 (2.161–14.901)		9.520 (2.947–30.755)	

* *p* < 0.05; *** *p* < 0.001. ADDwoR, anterior disc displacement without reduction.

**Table 4:** Correlation of the condylar bone resorption score with electrophysiological abnormalities.

	Electromyography Condition			
Characteristic	Crude OR (95%CI)	*p* Value	Adjusted OR (95%CI)	*p* Value
Age (y)	1.027 (0.958–1.102)	0.450	0.972 (0.903–1.047)	0.456
Sex				
Male	1.00 (reference)	0.358	1.00 (reference)	0.255
Female	1.597 (0.589–4.333)		1.930 (0.622–5.989)	
Condylar resorption score	2.235 (1.319–3.788)	0.003 **	2.112 (1.227–3.636)	0.007 **

** *p* < 0.01. ADDwoR, anterior disc displacement without reduction.

**Table 5:** Exploratory analysis of disc morphology score associated with electrophysiological abnormalities.

	Electromyography Condition			
Characteristic	Crude OR (95%CI)	*p* Value	Adjusted OR (95%CI)	*p* Value
Age (y)	1.027 (0.958–1.102)	0.450	0.966 (0.897–1.039)	0.428
Sex				
Male	1.00 (reference)	0.358	1.00 (reference)	0.351
Female	1.597 (0.589–4.333)		1.555 (0.522–4.635)	
Disc morphology score	1.243 (0.847–1.823)	0.267	1.343 (0.893–2.020)	0.156

## Data Availability

All data generated or analyzed during this study are included in this published article.

## Supplementary Materials

The following supporting information can be downloaded at: https://www.mdpi.com/article/10.3390/jcm15135148/s1, Figure S1: Flow diagram of participant selection; Table S1: Association between condylar bone resorption grades with electromyography condition; Table S2: Association between disc morphology subtypes and electromyography condition in female; Table S3: Correlation of disc position with electromyography condition in female; Table S4: Correlation of the condylar bone resorption score with electromyography condition in female; Table S5: Correlation of disc morphology score with electromyography condition in female; Table S6: FDR adjusted q values for predictors; Table S7: FDR adjusted q values for predictors in the three logistic regression models.

## Abbreviations

The following abbreviations are used in this manuscript:

TMJ	Temporomandibular joint
TMD	Temporomandibular disorder
ADD	Anterior disc displacement
ADDwoR	Anterior disc displacement without reduction
sEMG	Surface electromyography
nEMG	Needle electromyography
MUP	Motor unit potential
