# Supplementary material for: Exploration of the Association Between Anterior Disc Displacement Without Reduction and Electromyographic Activity of Masseter Muscle Based on Needle Electromyography
Source: J Clin Med. 2026 Jul 2;15(13):5148. doi: 10.3390/jcm15135148 (PMC13363582; doi:10.3390/jcm15135148)
Supplement: Supplementary file 1 [file jcm-15-05148-s001.zip › jcm-4329592-supplementary.pdf]

Supplementary Table S1. Association between condylar bone resorption grades with electromyography condition

Characteristic	Electromyography condition			
	Crude OR (95%CI)	*P value*	*Adjusted OR (95%CI)*	*P value*
Age (y)	1.027 (0.958-1.102)	0.450	1.033 (0.957-1.116)	0.402
Sex				
Male	1.00 (reference)	0.358	1.00 (reference)	0.028*
Female	1.597 (0.589-4.333)		4.347 (1.171-16.131)	
Degree of condylar resorption				
Control	1.00 (reference)		1.00 (reference)	
ADDwoR/				
mild	5.577(1.944-15.669)	0.001**	9.482 (2.719-33.062)	<0.001***
severe	5.800 (1.923-17.493)	0.002**	9.571 (2.616-35.021)	<0.001***

*P < .05; **P< .01; ***P < .001

ADDwoR, anterior disc displacement without reduction.

Supplementary Table S2. Association between disc morphology subtypes and electromyography condition in female

Characteristic	Electromyography condition			
	Crude OR (95%CI)	*P value*	*Adjusted OR (95%CI)*	*P value*
Age (y)	1.027 (0.958-1.102)	0.450	1.025 (0.949-1.108)	0.530
Sex				
Male	1.00 (reference)	0.358	1.00 (reference)	0.029*
Female	1.597 (0.589-4.333)		4.309 (1.157-16.055)	
Disc morphology		0.004*		0.002*
Control	1.00 (reference)		1.00 (reference)	
ADDwoR/				
III	6.981 (2.318-21.025)	<0.001	10.815 (3.015-38.791)	<0.001***
IV	5.686 (1.805-17.912)	0.003	9.971 (2.591-38.372)	<0.001***
V	4.296 (1.011-18.260)	0.048	7.080 (1.427-35.119)	0.017***

*P < .05; **P< .01; ***P < .001

ADDwoR, anterior disc displacement without reduction

Supplementary Table S3. Correlation of disc position with electromyography condition in female

	Electromyography condition			
**Characteristic**	*Crude OR (95%CI)*	*P value*	*Adjusted OR (95%CI)*	*P value*
Age (y)	0.998 (0.923-1.072)	0.967	1.003 (0.923-1.089)	0.949
Disc position				
Control	1.00 (reference)	0.002**	1.00 (reference)	0.002**
ADDwoR	7.496 (2.099-26.770)		7.504(2.100-26.807)	

**P< .01

ADDwoR, anterior disc displacement without reduction.

Supplementary Table S4. Correlation of the condylar bone resorption score with electromyography condition in female

	Electromyography condition			
**Characteristic**	*Crude OR (95%CI)*	*P value*	*Adjusted OR (95%CI)*	*P value*
Age (y)	0.998 (0.923-1.072)	0.967	0.982 (0.908-1.061)	0.642
Degree of condylar resorption	1.684 (0.949-2.989)	0.075	1.686 (0.950-2.002)	0.074

**P< .01

ADDwoR, anterior disc displacement without reduction.

Supplementary Table S5. Correlation of disc morphology score with electromyography condition in female

	Electromyography condition			
**Characteristic**	*Crude OR (95%CI)*	*P value*	*Adjusted OR (95%CI)*	*P value*

Age (y)	0.998 (0.923-1.072)	0.967	0.978 (0.904-1.058)	0.580
Disc morphology	1.124 (0.738-1.713)	0.586	1.151 (0.747-1.773)	0.524

**P< .01

ADDwoR, anterior disc displacement without reduction.

Supplementary Table S6. FDR-adjusted q-values for predictors.

Variables	*P value*	*Adjusted Q value*
Sex, female (%)	<0.001***	0.006**
Age (y)	0.513	0.513
EMG abnormality, n (%)	<0.001***	0.003**
Duration (ms)	<0.001***	0.002**
Amplitude (μV)	0.011*	0.0015**
Area (μV×ms)	<0.001***	0.0132*

Supplementary Table S7. FDR-adjusted q-values for predictors in the three logistic regression models

Model 1			*Model 2*			*Model 3*		
Variables	*P value*	*Adjusted Q value*	*Variables*	*P value*	*Adjusted Q value*	*Variables*	*P value*	*Adjusted Q value*
Sex	0.028	0.042	Sex	0.456	0.456	Sex	0.428	0.428
Age (y)	0.397	0.397	Age (y)	0.255	0.383	Age (y)	0.351	0.428
Disc position	<0.001	0.003	Condylar resorption	0.003	0.021	Disc morphology	0.156	0.428

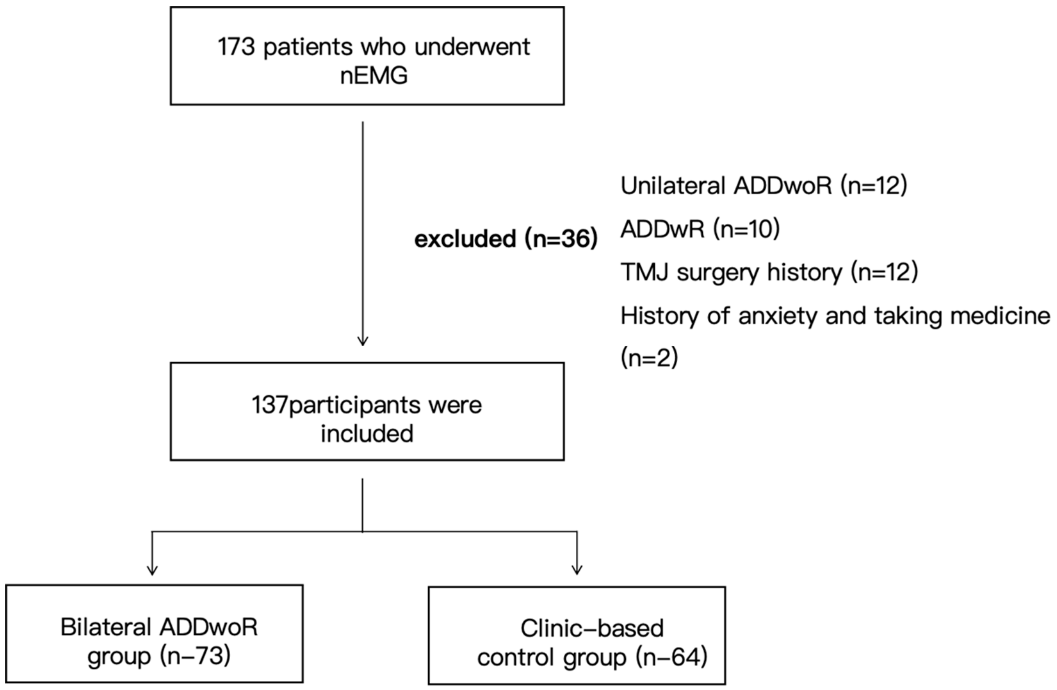

Supplementary Figure S1. Flow diagram of participant selection. Of 173 patients initially assessed, 36 were excluded (12 for unilateral ADDwoR; 10 for ADDwR; 12 for TMJ surgery history and 2 for history of anxiety and taking medication for intervention). The final analysis included 73 patients with bilateral ADDwoR and 64 clinic-based controls with normal TMJs.
